# A CRISPR/Cas9 genome editing pipeline in the EndoC-βH1 cell line to study genes implicated in beta cell function

**DOI:** 10.12688/wellcomeopenres.15447.2

**Published:** 2020-04-29

**Authors:** Antje K. Grotz, Fernando Abaitua, Elena Navarro-Guerrero, Benoit Hastoy, Daniel Ebner, Anna L. Gloyn

**Affiliations:** 1 Oxford Centre for Diabetes, Endocrinology & Metabolism, University of Oxford, Oxford, OX3 7LE, UK; 2 Wellcome Centre for Human Genetics, University of Oxford, Oxford, OX3 7BN, UK; 3 Target Discovery Institute, University of Oxford, Oxford, OX3 7FZ, UK; 4 Oxford NIHR Biomedical Research Centre, Churchill Hospital, Oxford, OX3 7LE, UK

**Keywords:** EndoC-βH1, human beta cells, CRISPR/Cas9, gene knockout, Type 2 Diabetes, insulin secretion, NEUROD1

## Abstract

Type 2 diabetes (T2D) is a global pandemic with a strong genetic component, but most causal genes influencing the disease risk remain unknown. It is clear, however, that the pancreatic beta cell is central to T2D pathogenesis. *In vitro* gene-knockout (KO) models to study T2D risk genes have so far focused on rodent beta cells. However, there are important structural and functional differences between rodent and human beta cell lines. With that in mind, we have developed a robust pipeline to create a stable CRISPR/Cas9 KO in an authentic human beta cell line (EndoC-βH1). The KO pipeline consists of a dual lentiviral sgRNA strategy and we targeted three genes ( *INS*, *IDE*, *PAM*) as a proof of concept. We achieved a significant reduction in mRNA levels and complete protein depletion of all target genes. Using this dual sgRNA strategy, up to 94 kb DNA were cut out of the target genes and the editing efficiency of each sgRNA exceeded >87.5%. Sequencing of off-targets showed no unspecific editing. Most importantly, the pipeline did not affect the glucose-responsive insulin secretion of the cells. Interestingly, comparison of KO cell lines for *NEUROD1* and *SLC30A8* with siRNA-mediated knockdown (KD) approaches demonstrate phenotypic differences. *NEUROD1-*KO cells were not viable and displayed elevated markers for ER stress and apoptosis. *NEUROD1*-KD, however, only had a modest elevation, by 34%, in the pro-apoptotic transcription factor CHOP and a gene expression profile indicative of chronic ER stress without evidence of elevated cell death. On the other hand, *SLC30A8*-KO cells demonstrated no reduction in K _ATP_ channel gene expression in contrast to siRNA silencing. Overall, this strategy to efficiently create stable KO in the human beta cell line EndoC-βH1 will allow for a better understanding of genes involved in beta cell dysfunction, their underlying functional mechanisms and T2D pathogenesis.

## Introduction

Type 2 diabetes (T2D) affects around 400 million people worldwide and is a complex disease with genetic and non-genetic risk factors ^1^. Genome-wide association studies (GWAS) have so far identified more than 240 loci which are robustly associated with disease risk ^2– 5^. The vast majority of these exert their impact on T2D-risk through the pancreatic beta cell and therefore authentic human beta cell models are essential for functional follow-up studies ^6^.

A lack of a stable and functional human beta cell line, restricted access to human cadaveric pancreatic islets and functional limitations of induced pluripotent stem cells (iPSC)-derived beta-like cells have long been a challenge in understanding beta cell biology. Meanwhile, rodent beta cell lines have provided valuable insights into beta cell function and pathophysiology ^7– 9^. Although they share many similarities with human beta cells, there are also fundamental structural, transcriptional and functional differences, aside from having a distinctive genetic background. Human pancreatic islets have a substantially different architecture than rodent islets as they have fewer beta cells, a mixed cell distribution throughout the whole islet, with alpha, beta and delta cells being adjacent to each other and alpha cells clustering around blood vessels ^10, 11^. Rodent islets on the other hand have a higher vascular density and are made up of a distinct beta cell core and non-beta cell mantle ^10, 12^. Transcriptomic analysis in purified beta cells from mice and human demonstrated a set of common core beta cell genes ^13, 14^. However, the studies also highlighted a substantial number of uniquely expressed genes in either species and significantly differentially expressed genes such as *GAD2, IAPP, MAFB* and *PPARG*, some of which are involved in Type 1 (T1D) and T2D pathology ^13– 15^. Differences in key components of the glucose-stimulated insulin secretion (GSIS) pathway emphasise unique functional signatures ^16^. The principal glucose transporter in rodent beta cells is *SLC2A2*, whereas human beta cells mainly utilise *SLC2A1* and *SLC2A3*, leading to distinct glucose uptake dynamics ^17– 19^. Furthermore, rodent beta cells express two insulin genes, *INS1* and *INS2*, which is in contrast to human beta cells, which only have one insulin gene ^20^. Other distinguishing factors are the proliferative capacity and ion channel composition of rodent cells compared to human beta cells ^21– 25^.

In 2011, Scharfmann and colleagues released the EndoC-βH1 cell line, a human beta cell line which opened up the possibility of studying human beta cell physiology and pathology *in vitro* and provided a valuable alternative to rodent beta cell lines ^26^. To generate this cell line, fetal pancreatic buds were transduced with oncogene simian virus 40 large tumour antigen (SV40LT) and human telomerase reverse transcriptase (hTERT). Between each transduction, the cells were transplanted into SCID mice to expand and form insulinomas. The isolated and passaged cells were able to secrete insulin in response to different glucose and secretagogues stimulation, expressed key beta cell markers and were negative for other pancreatic cell markers like glucagon ^26^. Insulin content is a magnitude lower than in primary human beta cells, but secreted insulin as percentage of content and the stimulation index are in the same range as for pancreatic islets ^16, 27^. Multiomic profiling in EndoC-βH1 cells including epigenomic and transcriptomic maps largely recapitulate primary human islets signatures and along with their similar electrophysiological properties, EndoC-βH1 are therefore a representative model of human beta cells and physiological insulin secretion ^28– 30^. Further independent investigations have also demonstrated their suitability for both high-throughput screening ^31, 32^ and individual gene function studies ^33– 35^.

Robust protocols for generating gene KO using CRISPR/Cas9 in EndoC-βH1 studies have not yet been described. This genome editing tool has revolutionised genetic manipulations by being an easily programmable RNA-guided endonuclease ^36– 39^. The application in EndoC-βH1, however, is not straightforward, as their proliferation rate is low and they are very sensitive to seeding densities. It is thus not possible to expand a culture from a single cell, which precludes the generation of a modified clonal cell line. Furthermore, the cells have a very low transfection efficiency, batch-to-batch variation and have to be closely monitored across passages to ensure their beta cells characteristics. A recent study created an *HNF1A* KO cell line in EndoC-βH1 using CRISPR/Cas9, this cell line, however, does not demonstrate complete *HNF1A* depletion and has not been fully characterised ^40^.

Despite the technical challenges of this cell line, we have successfully developed a lentiviral-based pipeline to create stable non-clonal CRISPR/Cas9 KO cell lines in EndoC-βH1 and have performed genomic and functional characterisation for several proof of concept genes. This CRISPR/Cas9 pipeline and its resulting KO cell lines could be a valuable tool in understanding human beta cell function and genes underlying the pathology for both T2D and T1D.

## Methods

### Cloning of individual sgRNA into plentiCRISPRv2

plentiCRISPRv2 was purchased from Addgene (#52961) ^41^ and sgRNA sequences were retrieved from the TKO Library v3 ^42^. Two sgRNAs per gene were chosen based on highest specificity and lowest off-target score which were evaluated on CRISPOR.org ^43^ ( Table 1). BsmBI compatible tails, 5’CACCGX3’ and 5’AAACYC3’, with X and Y being complementary sequences to the sgRNA, were added to each oligonucleotide. plentiCRISPRv2 vector was digested with FastDigest BsmBI (Fermentas) for 30 min at 37°C and gel-purified using a 0.8% agarose gel. sgRNA oligos were annealed (1 μl of each 100 μM stock) and phosphorylated using T4 PNK (NEB) for 30 min at 37°C, 5 min at 95°C, then the heating block was shut off to let the samples could cool down to room temperature (RT). 20 ng BsmBI digested plentiCRISPRv2 and 2 μl of 1:100 diluted annealed sgRNA oligonucleotides were ligated using Quick Ligase (NEB) for 1 h at RT. Next, 5 μl of the ligation reaction were transformed into Stbl3 competent cells and successful sgRNA insertion was confirmed using Sanger sequencing.

**Table 1.** sgRNA sequences for target genes.

sgRNA	Target gene	Target region	Sequence (5’ → 3’)
sgRNA 1	PAM	Exon 1	GAACTAGCAGGCTAGGGACG
sgRNA 2	PAM	Exon 12	GTTCAGAACCATACCACCAG
sgRNA 3	IDE	Exon 1	TACCCACACAGGCGCTCCGG
sgRNA 4	IDE	Exon 8	CATTAATGTGGACTTGACCG
sgRNA 5	INS	Exon 2	CACAATGCCACGCTTCTGCA
sgRNA 6	INS	Exon 2	CATCTGCTCCCTCTACCAGC
sgRNA 7	NEUROD1	Exon 2	CTTGCAAAGCGTCTGAACGA
sgRNA 8	NEUROD1	Exon 2	GCTGCGCTGTAGGCGTGCGG
sgRNA 9	SLC30A8	Exon 2	GTGTCCCAGAGAGAGACCAG
sgRNA 10	SLC30A8	Exon 8	GCACTCACTCACCATTCAGA

### Cell culture

EndoC-βH1 cells were cultured as previously described and passaged every 7 days ^26^. They were grown in culture vessels coated with 2 µg/ml Fibronectin and 1% extracellular matrix (ECM) (Sigma-Aldrich) and cultured in DMEM containing 5.5 mM glucose (Gibco), 2% bovine serum albumin (BSA), 2 mM glutamine, 10 mM nicotinamide, 100 international units (U)/ml penicillin, 100 µg/ml streptomycin (P/S), 50 μM β-2-mercaptoethanol, 5.5 μg/ml transferrin and 6.6 ng/ml sodium selenite (all Sigma-Aldrich).

Lenti-X HEK293T cells (Clontech) were cultured in DMEM 6429 (Sigma-Aldrich) containing 10% fetal calf serum, 100 U/ml penicillin and 100 µg/ml streptomycin. All cells were tested negative for mycoplasma and grown at 37°C and 5% CO _2_.

### Lentiviral production

Lenti-X HEK293T cells were grown to 80% confluency in T175 flasks and co-transfected with lentiviral packaging vectors in P/S free media. The transfection mix consisted of pMD2.G (6.85 μg) (Addgene #12259), psPAX2 (10.3 μg) (Addgene #12260), the respective cloned plentiCRISPRv2 (12.85 μg), 2 ml of JetPrime buffer and 60 μl of JetPrime transfection reagent (Polyplus transfection) per flask. After 15 min incubation at RT, the transfection mix was added to the cells and media was replaced after 16 h into fresh complete culture media. Supernatant containing viral particles was collected 48 h after transfection, spun down for 5 min at 2000 rpm and filtered through a 0.45-μm filter. Supernatant was ultracentrifuged for 2 h at 4°C and 29000 rpm in a swinging-bucket rotor. The virus pellet was resuspended in 1.5% BSA in PBS, aliquoted and stored at -80°C.

### Functional lentiviral titer

At 48 h before transduction, EndoC-βH1 were plated at 20,000 cells per well in a 96-well plate. A viral dilution curve ranging from 1:50 to 1:6400 was prepared in 100μl P/S free media and the cells were infected for 6 h. After 48 h, media was changed on half of the wells per dilution into 4 μg/μl puromycin and the cells were incubated for 7 days. Cell viability was analysed using the CyQUANT Direct Cell Proliferation assay (Invitrogen). The puromycin selected cell counts were normalised to their respective non-selected controls to determine the percentage of survival, which represents transduced cells. The functional titer in transducing units (TU)/μl can be calculated based on:

(1)

$$\text{TU}/\mu \text{l =}\;\frac{\text{\# Cells}\times \;m}{\text{Virus (}\mu \text{l) used in transduction}}$$

The probability that a cell is infected by a certain number of viral particles at a given multiplicity of infection (MOI) ( *m*) can be modelled using the Poisson distribution (PD). Simplifying the original PD equation, gives the following:

(2)

$$P(n>0)=1-e^{-m}$$

with *P*( *n*>0) being the probability that a cell gets infected by at least one viral particle ^44^. A MOI of 0.3 would lead to ~26% transduced cells, most of them being infected by a single viral particle, therefore this MOI is a good constant in determining the functional titer. To determine the MOI relative to the virus (μl) used in transduction in Equation 1, a linear regression for the percentage of alive cells against the amount of infected virus was performed in the linear, unsaturated range of the puromycin selection curve. The amount of virus needed for a MOI of 0.3 was then calculated by inserting 26% as the percentage of alive cells and solving the linear equation for the amount of virus (μl) needed in the transduction. Along with the number of plated cells, the TU/μl could then be determined.

### Generation of EndoC-βH1 KO cell lines

To generate stable CRISPR KO lines, cells were transduced at a MOI of 8 which was calculated based on the functional titer. They were selected in 4 μg/μl puromycin for 7 days, with media changes if necessary, to remove dead cells and add fresh puromycin. After selection, cells were grown in normal EndoC-βH1 culture medium and passaged weekly.

### Insulin secretion assay

Cells were starved overnight in 2.8 mM glucose followed by 30 min starvation in 0 mM glucose and stimulation for 1 h in one of the following conditions: 2.8 mM, 5.6 mM, 11 mM, 15 mM, 20 mM, 25 mM glucose, 15 mM glucose + 100 μM tolbutamide or diazoxide. All conditions were prepared in glucose-free EndoC-βH1 culture medium. Supernatant was collected and cells were lysed in acid ethanol to collect insulin content. Secreted and intracellular insulin were measured using the Insulin (human) AlphaLISA Detection Kit and the EnSpire Alpha Plate Reader (both Perkin Elmer), diluted 1:10 and 1:200, respectively. Secreted insulin was displayed as percentage of insulin content and insulin content was normalised to cell count, which was measured using the CyQUANT Direct Cell Proliferation assay (Invitrogen).

### siRNA silencing

Cells were transfected in 6-well plates at 24 h after plating with siRNAs at a final concentration of 15 nM (SMARTpool ON-TARGETplus human NeuroD1 #L-008667-00 and SLC30A8 #L-007529-01, non-targeted control pool #D-001810-10, Dharmacon). The required amount of siRNA (μM) (Gibco) was prepared in Opti-MEM reduced serum-free medium to constitute 0.1% of the volume. A Lipofectamine RNAiMAX (Invitrogen) mix was prepared to account for 0.4% of the same total volume and both transfection mixes were incubated for 5 min at RT. The RNAiMAX and siRNA mix were pooled and further incubated for 20 min before they were added dropwise to the cultured cells. Cells were harvested 72 h past transfection for RNA and protein extraction.

### Gene expression analyses

RNA was extracted using the TRIzol reagent (Invitrogen) following manufacturer’s instructions. First-strand cDNA was synthesised using the Super Script III First-Strand Synthesis System (Invitrogen), oligo(dT) primer and 50–500 ng total RNA as input. Quantitative PCR (qPCR) to measure gene expression levels were performed with TaqMan Gene Expression Assays and TaqMan Gene Expression Master Mix on a 7900HT (all Applied Biosystems) using the following thermocycling conditions: 2 min at 50°C, 10 min at 95°C and 40 cycles of 15 sec at 95°C and 1 min at 60°C ( Table 2). TaqMan probes with binding sites outside of the regions targeted by sgRNAs were used. Ct values were analysed using the ΔΔCt method and target genes were normalised to three housekeeping genes ( *TBP*, *PPIA* and *GAPDH*).

**Table 2.** TaqMan gene expression assays.

TaqMan Probe	Assay details	Target region
*INS*	Hs00355773_m1	Exon 1–2
*IDE*	Hs00610452_m1	Exon 24–25
*PAM*	Hs01084034_m1	Exon 22–23
*NEUROD1*	Hs01922995_s1	Exon 2
*GAPDH*	Hs02786624_g1	Exon 8
*TBP*	Hs00427620_m1	Exon 3–4
*PPIA*	Hs01634221_s1	Exon 1
*SLC30A8*	Hs00545182_m1	Exon 2–3
*DDIT3*	Hs99999172_m1	Exon 1–2
*XBP1s*	Hs03929085_g1	Exon 5
*HSPA5*	Hs00607129_gH	Exon 1–2
*ATF6*	Hs00232586_m1	Exon 6–7
*ATF4*	Hs00909569_g1	Exon 1–2

### Western blot analyses

Cell pellets for protein analyses were lysed in RIPA buffer (50 mM Tris pH 7.4, 150 mM NaCl, 1% Triton X-100, 0.5% sodium deoxycholate, 0.1% SDS) containing 1x protease inhibitor cocktail (Roche). Protein concentration was quantified by DC protein assay (Bio-Rad) and 10 μg of protein per lane were prepared. Lysates were denatured at 80°C for 10 min and run on a Mini-PROTEAN TGX 4–20% precast gel (Bio-Rad) at 300 V for 15 min. The gel was activated on a ChemiDoc MP Imaging System and transferred to a Trans-Blot Turbo polyvinylidene fluoride (PVDF) membrane using the Trans-Blot Turbo Transfer System (all Bio-Rad). Membranes were blocked in 3% BSA for 1 h at RT, incubated with primary antibodies overnight at 4°C followed by a 1 h incubation at RT with secondary antibodies (antibodies are given in Table 3). The membranes were subsequently incubated for 4 min at RT with Clarity Western enhanced chemiluminescence (ECL) reagent and imaged on the ChemiDoc MP Imaging System (Bio-Rad). To normalise for protein loading, the membrane was further incubated with a loading control antibody of appropriate size (tubulin or GAPDH) ( Table 3). Western Blot images were quantified using Image Lab 6.0 software (Bio-Rad). Protein bands of interest were normalised to a loading control on the same blot and displayed relative to a control sample.

**Table 3.** Antibody specifications.

Antibody	Company, catalogue number	Dilution	Species	RRID/Ref
INS	Santa Cruz, sc-377071	1:1000	Mouse monoclonal	AB_2800506
IDE	Santa Cruz, sc-393887	1:1000	Mouse monoclonal	AB_2800507
PAM	Santa Cruz, sc-514110	1:1000	Mouse monoclonal	AB_2800508
NEUROD1	Santa Cruz, sc-46684	1:1000	Mouse monoclonal	AB_671759
Cas9	Santa Cruz, sc-517386	1:1000	Mouse monoclonal	AB_2800509
CHOP	Abcam, ab179823	1:1000	Rabbit monoclonal	AB_10703186
pPERK	Cell Signaling, #3179	1:1000	Rabbit monoclonal	AB_2095853
pIRE1	Abcam, ab48187	1:1000	Rabbit polyclonal	AB_873899
Cleaved Caspase 3	Cell Signaling, #9661	1:500	Rabbit polyclonal	AB_2341188
ZnT8	/	1:1000	Mouse monoclonal	45
β-Tubulin	Santa Cruz, sc-365791	1:2000	Mouse monoclonal	AB_10841919
GAPDH	Abcam, ab181602	1:10 000	Rabbit monoclonal	AB_2630358
α-mouse IgG HRP	Thermo Scientific, 31450	1:2500	Rabbit polyclonal	AB_228427
α-rabbit IgG HRP	Thermo Scientific, 31460	1:2500	Goat polyclonal	AB_228341

### PCR and sequencing analyses

Genomic DNA for PCR amplification was extracted using the NucleoSpin Tissue extraction kit (Macherey-Nagel). PCR reactions were prepared containing the following components per sample: 2 µl Immobuffer (10x), 0.6 µl MgCl _2_ (50 mM), 1 µl each of forward and reverse primer (10 µM) ( Table 4), 0.4 µl dNTPs (10 mM), 0.2 µl Immolase DNA Polymerase (5 U/µl) (all Bioline), 4 µl Q-Solution (5x) (Qiagen) and 10.8 µl of the DNA sample (100 ng). The PCR amplification was performed for 10 min at 94°C, 32 cycles of 1 min at 94°C, 1 min at 64°C and 30 sec at 72°C, followed by 10 min at 72°C. DNA samples were run on a 2% agarose gel for 1 h at 120 V and PCR bands were visualised on a GelDoc transilluminator system (Bio-rad). Ahead of sending samples for sequencing, excess primers and nucleotides were removed from PCR reactions using an ExoSAP enzymatic clean up. The reaction was performed with 10 µl PCR product, 0.05 µl ExoI, 0.5 µl SAP, 1 µl SAP buffer (all Affymetrix) and 0.45 µl nuclease-free water and incubated for 30 min at 37°C and 5 min at 95°C. The sequencing reaction was premixed using 13.5 µl nuclease-free water, 1.5 µl ExoSAP treated PCR sample and 2 µl sequencing primer (10 µM) and sent to Eurofins Genomics. Sequence traces were visualised using SnapGene Viewer 4.3 and analysed using TIDE 2.0 and ICE 1.1 ^46, 47^.

**Table 4.** Primer specifications.

Primer name	Target region	Experiment	Sequence (5’ → 3’)
LKO1.5R	lentiCRISPRv2	sgRNA sequence	GTTGATAACGGACTAGCCT
lentiCRv2_Cas9	*Cas9*	sgRNA integration	CAGGCCGATGCTGTACTTCT
PAM_sgRNA1	sgRNA1	sgRNA integration	ACACCGGAACTAGCAGGCTA
PAM_sgRNA2	sgRNA2	sgRNA integration	GACGAAACACCGGTTCAGAAC
IDE_sgRNA3	sgRNA3	sgRNA integration	AAACACCGTACCCACACAGG
IDE_sgRNA4	sgRNA4	sgRNA integration	CGCATTAATGTGGACTTGACCG
INS_sgRNA5	sgRNA5	sgRNA integration	CAATGCCACGCTTCTGCAG
INS_sgRNA6	sgRNA6	sgRNA integration	CATCTGCTCCCTCTACCAGC
NEUROD1_sgRNA7	sgRNA 7	sgRNA integration	GACGAAACACCGCTTGCAAA
NEUROD1_sgRNA8	sgRNA 8	sgRNA integration	CTGTAGGCGTGCGGGTTTT
PAM1_F	sgRNA1 target site	Editing efficiency	GCTGGAGGGAGGAAAGCTTC
PAM1_R	sgRNA1 target site	Editing efficiency	TTTTTCTGCACGGGGGACTT
PAM2_F	sgRNA2 target site	Editing efficiency	TTGCTGGCAGATCTAAGGGC
PAM2_R	sgRNA2 target site	Editing efficiency	TCCCTGGCTGAGATTTTCCTC
IDE3_F	sgRNA3 target site	Editing efficiency	AGTCGCCGGATTCCTTTACC
IDE3_R	sgRNA3 target site	Editing efficiency	CTAATGCGGTACCGGCTAGC
IDE4_F	sgRNA4 target site	Editing efficiency	TCCATGAAACAAAGGCCAAGT
IDE4_R	sgRNA4 target site	Editing efficiency	CCCCACTTCTGCACCATCTT
INS5_F	sgRNA5 target site	Editing efficiency	CATCTCTCTCGGTGCAGGAG
INS5_R	sgRNA5 target site	Editing efficiency	TCCCTCTAACCTGGGTCCAG
INS6_F	sgRNA6 target site	Editing efficiency	CCTGTAGGTCCACACCCAGT
INS6_R	sgRNA6 target site	Editing efficiency	AAGACACACAGACGGCACAG
sgRNA1_MED15_F	sgRNA1 off-target	Off-targets	GGCCAAACACACAGAGGAGT
sgRNA1_MED15_R	sgRNA1 off-target	Off-targets	TGGACTTGCCCTCTCTTGAC
sgRNA2_GPM6B_F	sgRNA2 off-target	Off-targets	ATCACTGCAGGGAACTGCTT
sgRNA2_GPM6B_R	sgRNA2 off-target	Off-targets	CAGCACCATCCTCAGATCCT

### Statistical analysis

Statistical analyses were performed in Prism 8.0 (GraphPad Software) and data are shown as mean with standard error of the mean (SEM). Values displayed as fold changes were analysed as log-transformed values and statistical tests were performed as indicated in the figure legends. In general, values normalised to a control group such as western blot data were analysed using one-sample Student’s t-test and two or more groups were compared using two-sample Student’s t-test or one-way analysis of variance (ANOVA) followed by Sidak’s multiple comparison test.

## Results

### A CRISPR/Cas9 pipeline to create EndoC-βH1 KO cells

**Figure 1.**
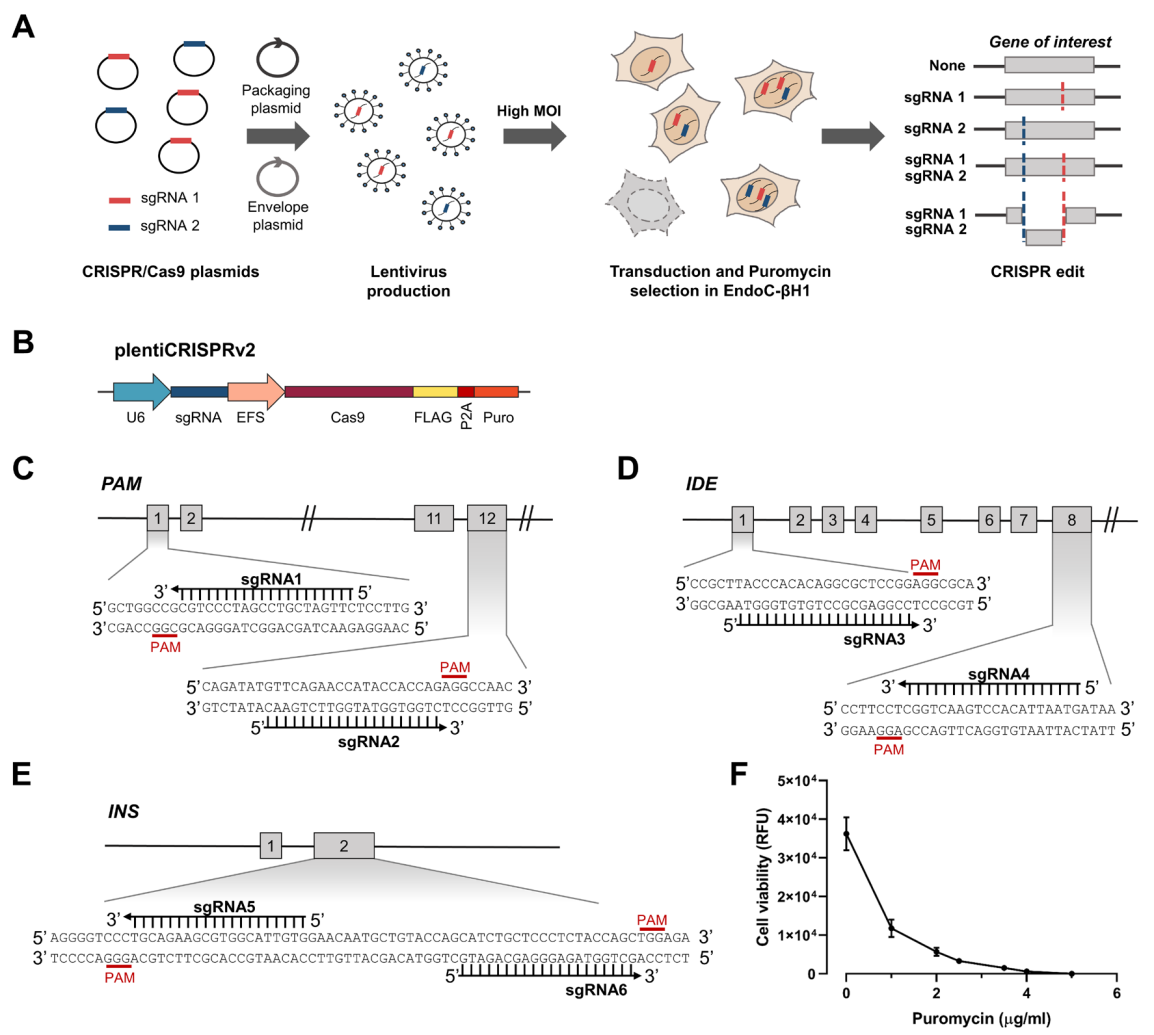
A lentiviral CRISPR/Cas9 pipeline to genome edit EndoC-βH1. ( **A**) Strategy to genome edit EndoC-βH1 using a lentiviral dual sgRNA-based approach resulting in a population with cells containing individual or both sgRNA edits. ( **B**) CRISPR plasmid, lentiCRISPRv2 containing Cas9 and a single sgRNA. ( **C**– **E**) Gene targeting strategy using two sgRNA per gene for *PAM* ( **C**), *IDE* ( **D**) and *INS* ( **E**). Numbers indicate exons of the respective gene. ( **F**) Puromycin kill curve in EndoC-βH1. Relative fluorescence units (RFU) are representative for cell viability. Results are representative of the optimization experiment performed right before selection of the KO cell lines. The graph displays the mean of six technical replicates with standard error of the mean (SEM).

To demonstrate that this lentiviral CRISPR/Cas9 pipeline can robustly generate KOs in EndoC-βH1, we created KO cell lines for three proof-of-concept genes. We chose genes with known relevance in beta cell function, namely peptidyl-glycine alpha-amidating monooxygenase ( *PAM*) ^3, 33^, insulin-degrading enzyme ( *IDE*) ^48, 49^ and insulin ( *INS*) ^50, 51^. In brief, we transduced EndoC-βH1 with lentivirus containing Cas9 and two sgRNAs, selected for successfully transduced cells and characterised the generated heterogeneous KO cell lines ( Figure 1A). As a vector system, we chose lentiCRISPRv2 which is a one-plasmid system containing Cas9, a puromycin resistance cassette and the cloned sgRNA ( Figure 1B). To increase the KO efficiency in our editing approach, we utilised a dual sgRNA strategy using two sgRNAs in separate lentivirus targeting different parts or exons of each gene ( Figure 1C–E) ^52^. The sgRNA sequences were retrieved from the genome-wide CRISPR KO library Toronto KnockOut version 3.0 (TKOv3) which are optimized for high on-target efficiency and minimal off-target cutting ^42, 53^. In the case of *IDE*, only one of the two protein-coding isoforms was targeted. We packaged lentiCRISPRv2 into lentivirus and transduced EndoC-βH1 at a high MOI of 8, ensuring that each cell is infected by several lentivirus and increasing the likelihood to achieve KOs. The cells were selected in 4 µg/ml puromycin to remove untransduced cells. The ideal concentration of puromycin was determined right before selection as EndoC-βH1 cells have different susceptibilities to antibiotics depending on their passage ( Figure 1F). After antibiotic selection, the transduced cells are a heterogeneous population having either no edit, an insertion or deletion (indel) from one sgRNA, a large deletion from simultaneous cutting of both sgRNA or two indels from both sgRNA cutting individually ( Figure 1A). These stable CRISPR cell lines were routinely cultured like regular EndoC-βH1 cells and their genomic and functional characteristics investigated.

### Genomic modifications of EndoC-βH1 KO cells

**Figure 2.**
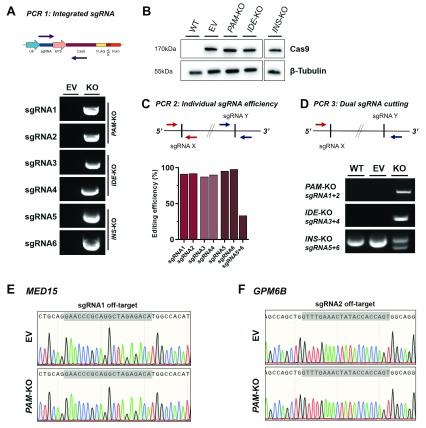
Genomic modifications of EndoC-βH1 KO cell lines. ( **A**) Stable genomic integration of lentiCRISPRv2 in transduced EndoC-βH1. *Upper panel:* PCR strategy (PCR 1) using primer specific for each sgRNA together with generic Cas9 primer. PCR product is only present upon successful integration of the specific sgRNA. *Lower panel:* PCR products for individual sgRNAs in control EV cell line or KO cell lines. ( **B**) Western blot analysis of Cas9 protein and β-Tubulin in KO cell lines and control cells (WT and EV). ( **C**) Editing efficiency of individual sgRNAs. *Upper panel:* PCR strategy (PCR 2) using primer specific for each sgRNA target site. *Lower panel:* PCR products for individual sgRNA target sites were Sanger sequenced and analysed for editing efficiency using TIDE and ICE. ( **D**) Editing efficiency of dual sgRNA cutting. *Upper panel:* PCR strategy (PCR 3) using primers flanking the region encompassed by both sgRNAs. PCR product (or shorter fragment) is only present upon deletion of the full region between both sgRNA target sites. *Lower panel:* PCR products indicating large deletion in WT, EV or KO cell lines. ( **E**, **F**) Sequencing of top off-target hits in *PAM*-KO cells. Sequencing trace for sgRNA1 off-target *MED15* ( **E**) and sgRNA2 off-target *GPM6B* ( **F**) are displayed for EV and *PAM*-KO cells.

To characterise the genomic modifications resulting from stable lentiCRISPRv2 integration and CRISPR editing, we analysed sgRNA and Cas9 integration, sgRNA efficiency and potential off-target cutting. A control cell line created with the same lentiviral backbone but without sgRNA was included as empty vector (EV) or Cas9 only control in all experiments. A PCR-based approach (PCR 1) using a sgRNA specific primer and a primer targeting the lentiCRISPRv2 backbone was used to detect sgRNA integration ( Figure 2A). Both sgRNAs were detectable in each KO cell line and not in EV control cells indicating successful transduction with both sgRNA lentivirus. Stable Cas9 expression was demonstrated in all cells lines transduced with lentiCRISPRv2 (KO cell lines and EV) but not in untransduced wild-type cells (WT) ( Figure 2B). Individual sgRNA editing efficiency was assessed by amplifying sgRNA target sides (PCR 2) and measuring indel frequency using TIDE ( Figure 2C) ^46^. All sgRNA target sides demonstrate an editing efficiency greater than 87.5%, leaving only around 1% of cells without indels. The sgRNA target sides in *INS*-KO cells (sgRNA 5 and 6) are within range of a single PCR and Sanger sequencing reaction, which makes it possible to assess the frequency of large deletions from simultaneous cutting of both sgRNA using the Inference of CRISPR Edits (ICE) tool ^47^. In 33.4% of *INS*-KO cells, the approximately 50 bp region between both sgRNA target sides has been deleted through concurrent sgRNA cutting ( Figure 2C). In *PAM*-KO and *IDE*-KO cells, the presence of large deletions between both sgRNAs was demonstrated by performing a PCR with primers on either side of the sgRNA target side (PCR 3) ( Figure 2D). If the region between the two sgRNAs is still present, the fragment is too large for PCR amplification but if both sgRNAs cut simultaneously and the region between the sgRNA target sides has been deleted, a PCR product can be amplified. Such PCR product is present in both cell lines, *PAM*-KO and *IDE*-KO, indicating a large deletion of 94 kbp and 66 kbp, respectively, that has not occurred in the WT and EV control cells. In *INS*-KO cells and confirming the results of the ICE analysis, the presence of two bands in contrast to controls indicate the presence of both fragments, a shorter PCR product containing the 50 bp deletion and the normal PCR product which results from cells containing small indels or no edits. Further, we tested if the stable integration of sgRNA and Cas9 in our CRISPR pipeline increased potential off-target activity. We determined the off-target potential by assessing the cutting frequency determination (CFD) score of sgRNA 1 and 2 off-targets in *PAM*-KO cells ^54^. The regions with the highest CFD score, introns in *MED15* (0.43) and *GPM6B* (0.54) were sequenced and no off-target activity could be detected ( Figure 2E and F). In summary, this CRISPR pipeline in EndoC-βH1 is highly efficient in creating edited populations containing individual indels or large deletions from two double-strand breaks.

### Functional characterisation of EndoC-βH1 KO cells

**Figure 3.**
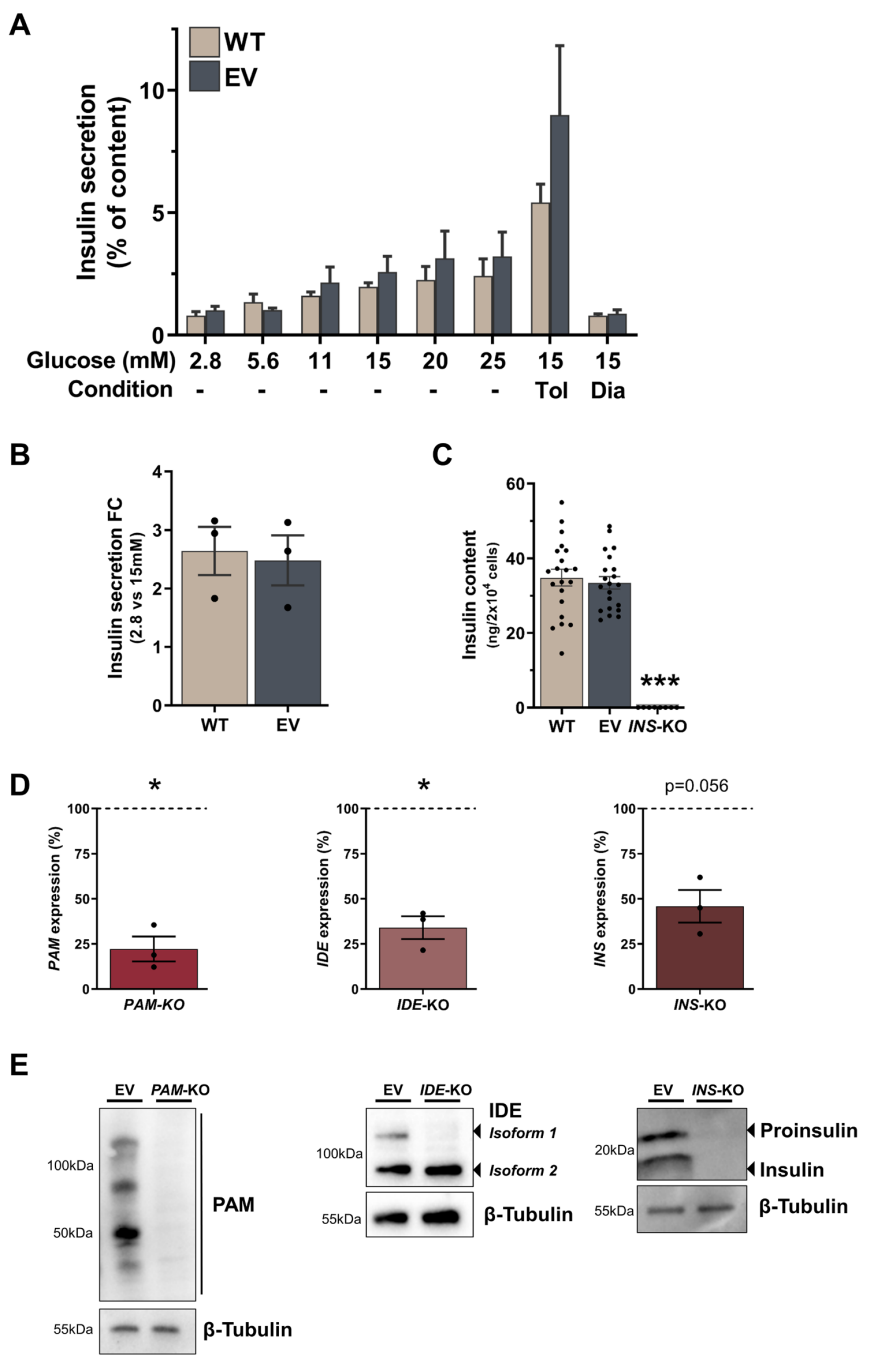
Functional characterisation of EndoC-βH1 KO cell lines. ( **A**– **C**) Insulin secretion as percentage of content in different glucose conditions ( **A**), fold change between 2.8 mM and 15 mM glucose stimulation ( **B**) and insulin content ( **C**) in WT cells compared to EV cells. ( **D**) qPCR expression data for *PAM, IDE* and *INS* genes in EV and respective KO cell lines. Values for KO cell lines were normalised to EV within each experiment and fold changes are displayed as percentage of EV, which is indicated as a dotted line at 100%. ( **E**) Western blot analysis of PAM, IDE and INS in EV and respective KO cells with β-Tubulin as loading control. All data are mean ± SEM from three independent experiments and for insulin content as 21 replicates from seven conditions in three independent experiments. Fold changes were log-transformed for statistical analysis. P values * < 0.05, ** < 0.01 and *** < 0.001 using two-way ANOVA Sidak’s multiple comparison test ( **A**), two-sample t-test with Holm-Sidak correction for Diazoxide and Tolbutamide induction and glucose stimulation ( **A, B**), one-way ANOVA with Sidak’s multiple comparison test for insulin content ( **C**) and one-sample t-test for expression data ( **D**).

Having established efficient editing of the cells, we next sought to determine if this translated into functional KO cells. We therefore investigated the insulin secretion characteristics of Cas9 expressing cells and determined both mRNA expression and protein levels of the targeted genes in the KO cells. To investigate if the transduction and selection pipeline or general expression of Cas9 affects the functionality of the cells, we compared EV to WT cells and assessed their insulin secretion and content characteristics. The secretory capacities of EV and WT cell lines in response to physiological glucose concentrations stimulation are similar ( Figure 3A). Both cell lines were susceptible to the ATP-sensitive potassium (K _ATP_) channel activator and blocker, diazoxide and tolbutamide. Diazoxide reduced insulin secretion by 66.2% and 59.8% (p=0.81), whereas tolbutamide further potentiated insulin secretion 3.5- and 2.8-fold (p=0.94) in WT and EV cells, respectively ( Figure 3A). Insulin secretion increased 2.48- vs 2.64-fold (p=0.94) in EV compared to WT cells after stimulation with 15 mM glucose ( Figure 3B). Intracellular insulin was equally similar between EV and WT cells, averaging at 33.44 ng and 34.83 ng/2×10 ^4^ cells (p=0.83) ( Figure 3C). mRNA levels of targeted genes were measured by RT-qPCR and were significantly decreased in *PAM*-KO and *IDE*-KO by 77.8% (p=0.035) and 66% (p=0.034), respectively. *INS*-KO cells also demonstrate a strong reduction of *INS* transcript by 54.2% (p=0.056) compared to EV control cells ( Figure 3D). These reductions of mRNA levels indicate the successful introduction of frameshift mutation and the generation of a premature stop codon (PSC) followed by degradation through nonsense-mediated mRNA decay (NMD). The detection of residual transcript is in line with previous studies demonstrating incomplete NMD in KO genes after induction of frameshift mutations in coding regions of the gene ^55^. Due to the heterogeneity of the KO cell population however, it cannot be ruled out that the detected transcript also includes mRNA from unedited cells or cells that do not contain a frameshift mutation or PSC. In *IDE*-KO cells, only one protein-coding isoform was targeted for CRISPR editing. However, the detected *IDE* transcript is representative for both isoforms, suggesting that part of the residual transcript originates from the non-targeted expressed isoform. In fact, when protein levels were assessed by western blot in *IDE*-KO, the targeted isoform (Isoform 1) was not detectable, whereas the non-targeted isoform demonstrates unchanged protein expression. In contrast, we observed complete depletion of PAM in *PAM*-KO and insulin precursor and mature insulin in *INS*-KO cells ( Figure 3E). In addition, when insulin content was assessed in *INS*-KO cells using a sensitive AlphaLISA-based method, insulin was not detectable within the dynamic range of the assay (p<0.0001 vs EV) ( Figure 3C). The complete absence of protein indicates that the created KO cell lines are all indeed loss-of-function (LoF) cell lines. The KO cell lines were stably cultured for more than six months without losing their KO phenotype. We can conclude that this lentiviral CRISPR pipeline in EndoC-βH1 creates functional KO cells with complete protein depletion and without any adverse effects on insulin secretion due to stable expression of Cas9 or antibiotic selection.

### EndoC-βH1 KO cells represent a distinct loss-of-function model

**Figure 4.**
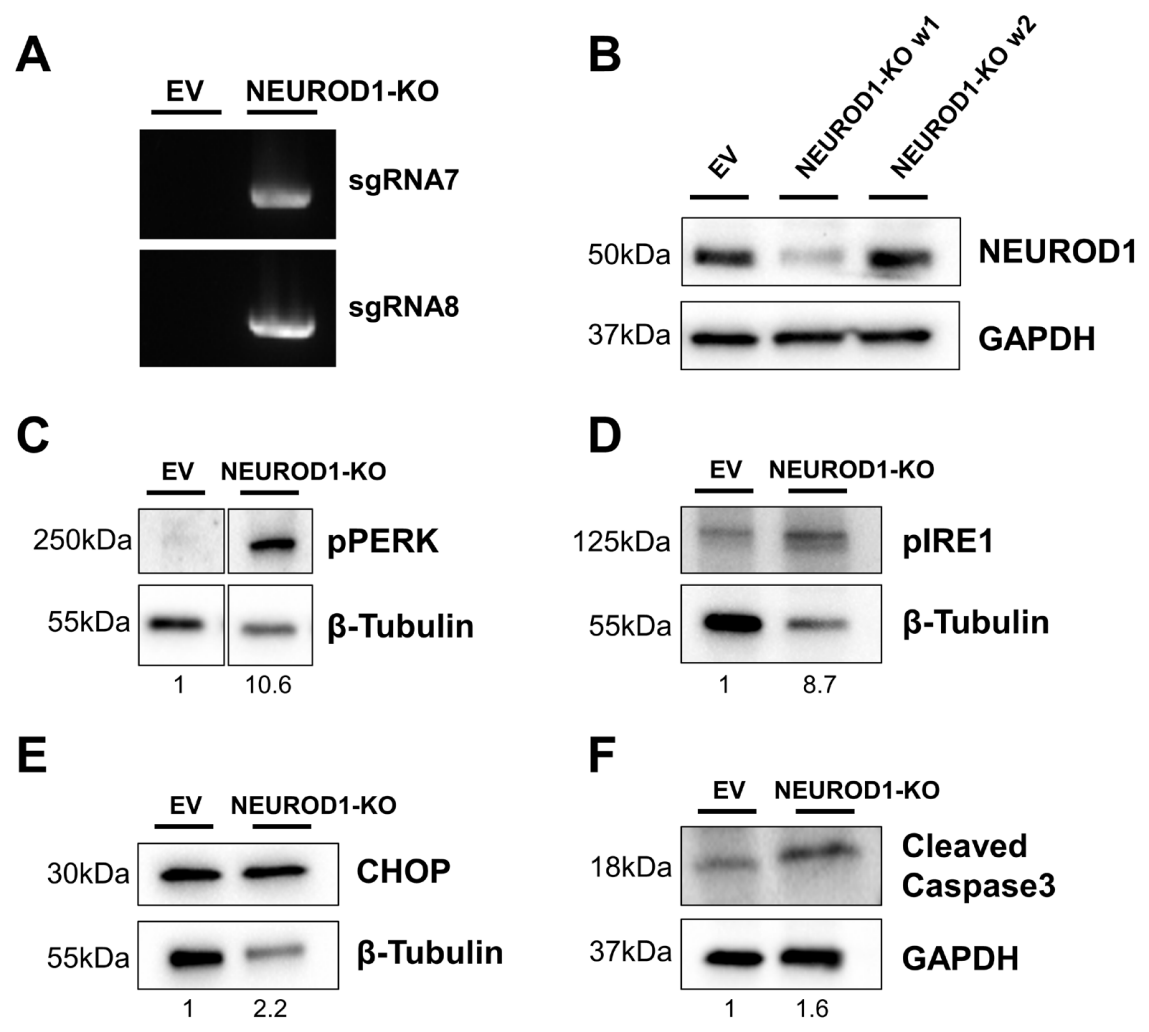
*NEUROD1*-KO cells demonstrate elevated ER stress and apoptosis. ( **A**) PCR product for lentiCRISPRv2 and NeuroD1 sgRNA in control EV and *NEUROD1*-KO cells as described in Figure 2A. ( **B**) Western Blot analysis for NEUROD1 expression in EV and *NEUROD1*-KO cells one week apart (w1 and w2). ( **C**– **F**) Western blot analysis in EV and *NEUROD1*-KO (w1) for pPERK ( **C**), pIRE1 ( **D**), CHOP ( **E**) and cleaved caspase 3 ( **F**). GAPDH or β-Tubulin are indicated as loading controls and data is from one *NEUROD1* KO cell line. Values below each lane represent the fold change of protein levels in *NEUROD1*-KO cells compared to EV cells.

**Figure 5.**
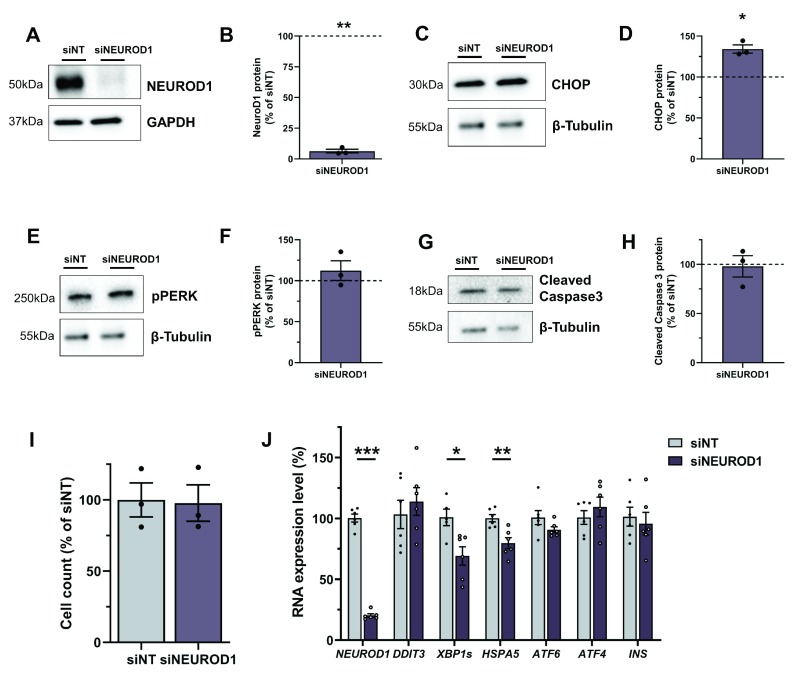
*NEUROD1* siRNA silencing results in increased CHOP and chronic ER stress. ( **A**– **H**) Western Blot analysis in cells treated with siRNA targeting *NEUROD1* (si *NEUROD1*) compared to control siRNA (siNT). Western blots and quantification for NEUROD1 ( **A**, **B**), CHOP ( **C**, **D**), pPERK ( **E**, **F**) and cleaved caspase 3 ( **G**, **H**), β-Tubulin and GAPDH are displayed as loading controls. Protein values were normalised to their respective loading controls and siNT within each experiment and fold changes are displayed as percentage of siNT, which is indicated as a dotted line at 100%. ( **I**) Cell count data for si *NEUROD1* cells is normalised to siNT. ( **J**) Expression data analysis for genes involved in ER stress in siNT and si *NEUROD1* cells. All data are mean ± SEM from three independent experiments for western blots and cell count and six independent experiments for expression data. Fold changes were log-transformed for statistical analysis. P values * < 0.05, ** < 0.01 and *** < 0.001 using one-sample t-test for western blot data ( **B**, **D**, **F**, **H**) and two-sample t-test for cell count ( **I**) and expression data ( **J**).

To assess EndoC-βH1 KO cells as a LoF model, we compared KO cells to siRNA silencing strategies. siRNA mediated effects are based on mRNA degradation and unlike stable KO cells, they represent a transient LoF system ^56^. To investigate the different strategies, we generated *SLC30A8*-KO and *NEUROD1*-KO cell lines and compared them with their respective siRNA models. *NEUROD1* is a key transcription factor for beta cell function and pancreas development and is implicated in both T2D-risk and monogenic diabetes ^57– 59^. The *NEUROD1*-KO cell line creation was successful as assessed by sgRNA integration ( Figure 4A). However, it was not possible to generate a stable KO cell line as *NEUROD1*-KO cells were not able to survive. Within a few passages and after an initial reduction in NEUROD1, the protein level returned to baseline indicating that *NEUROD1*-KO cells were depleted, and only unedited cells survived and expanded ( Figure 4B). Protein samples taken during the brief period of NEUROD1 reduction demonstrated an increased level of markers for endoplasmic reticulum (ER) stress and apoptosis. Unfolded protein response (UPR) signal activators, PERK and IRE1, which are activated through phosphorylation (pPERK and pIRE1) to initiate downstream signalling aimed at restoring ER homeostasis were increased 10.6- and 8.7-fold respectively ( Figure 4C and D). However, a 2.2-fold upregulation of the pro-apoptotic transcription factor CHOP and a 1.6-fold increase in the activated death protease cleaved caspase 3 indicated persistent and severe ER stress resulting in apoptosis in *NEUROD1*-KO cells ( Figure 4E and F). There was no difference in ER stress or apoptotic markers between WT and EV cells (not shown). To compare these severe consequences of NEUROD1 depletion to a transient model, we performed siRNA KD achieving a mean protein reduction of 93.7% (p=0.007) ( Figure 5A and B). CHOP, a downstream transcription factor mediating apoptosis was significantly upregulated by 34% (p=0.016) compared to non-targeting control siRNA ( Figure 5C and D). Other markers of UPR activation and apoptosis like pPERK (112.3%, p=0.422) and cleaved caspase 3 (98.0%, p=0.801) were not significantly increased ( Figure 5E–H and *Extended data*). A combined readout for cell death and proliferation does not show a difference between control and *NEUROD1* silenced cells (100% vs 97.78%, p=0.894), illustrating no apoptotic effects in cells treated with si *NEUROD1* ( Figure 5I). Analysis of expression levels in siRNA treated cells confirm efficient silencing of *NEUROD1* mRNA levels by 79.72% (p<0.0001) ( Figure 5J). *DDIT3*, which encodes for CHOP was in contrast to its protein level, not significantly increased (113.93%, p=0.506). The active form of *XBP1*, spliced *XBP1* ( *XBP1s*) is a downstream transcription factor inducing the expression of UPR target genes, which was significantly downregulated by 30.82% (p=0.023). *HSPA5*, encoding the ER chaperone BiP was also significantly decreased to 79.73% (p=0.005). There was also a trend towards *ATF6* reduction to 90.80% (p=0.143). *ATF4* and *INS* were not significantly changed (p=0.453 and p=0.596). This observed expression phenotype is consistent with an adaptive UPR response to chronic ER stress ^60^. The siRNA silencing approach confirms an effect of *NEUROD1* on ER stress; however, the phenotype is more pronounced and severe in the KO model. Whereas the silencing of *NEUROD1* does not have an apoptotic effect, complete loss of NEUROD1 protein in *NEUROD1*-KO cells leads to cell death.

**Figure 6.**
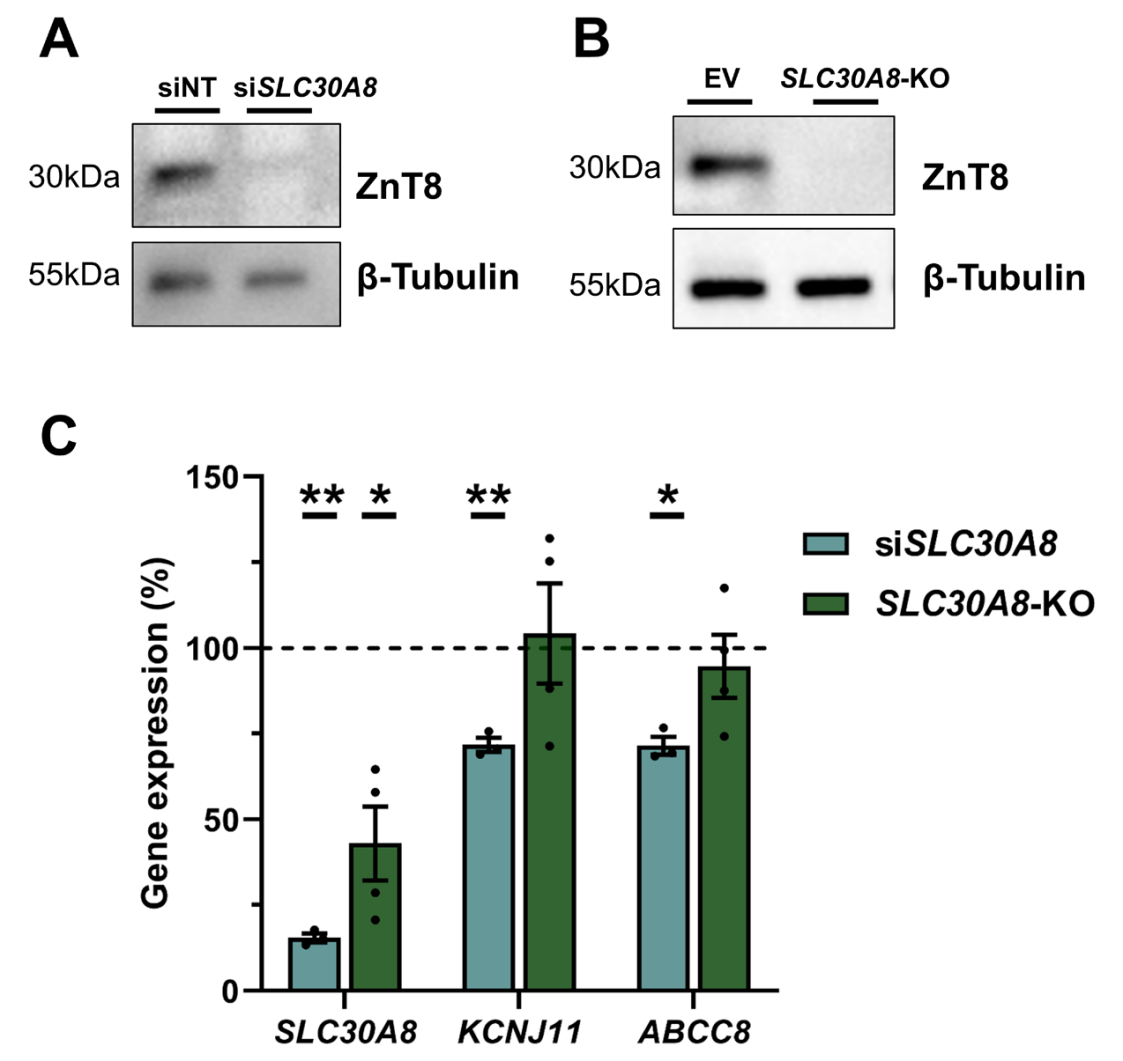
SLC30A8-KO cells do not replicate siRNA mediated effects on K _ATP_ channels. ( **A**, **B**) Western blot analysis for Znt8 in cells treated with si *SLC30A8* compared to control siNT ( **A**) and *SLC30A8*-KO cells compared to EV cells ( **B**). β-Tubulin is indicated as loading control. **C**, Gene expression analysis of *SLC30A8, KCNJ11* and *ABCC8*. *SLC30A8*-KO cells and si *SLC30A8* were normalised within each experiment to their respective control (EV or siNT) which are displayed as a dotted line at 100%. All data are mean ± SEM from three independent experiments and fold changes were log-transformed for statistical analysis. P values * < 0.05, ** < 0.01 and *** < 0.001 using one-sample t-test comparing treated cells to their control at 100%.

As a second comparison between the KO and siRNA model, we chose the *SLC30A8* gene, which encodes the zinc transporter ZnT8. LoF variants in *SLC30A8* have shown to be protective in T2D and siRNA KD of *SLC30A8* in EndoC-βH1 has been associated with improved glucose sensitivity and reduced expression of K _ATP_ channel subunits, amongst other effects ^34^ *.* To assess differences between the siRNA and KO model, we focused on comparing the expression of the K _ATP_ channel subunits, *KCNJ11* and *ABCC8*. siRNA silencing of *SLC30A8* reduced ZnT8 protein by 76.39% (p=0.001) and mRNA expression was decreased by 84.54% (p=0.002) ( Figure 6A and C). *SLC30A8*-KO cells demonstrate complete ZnT8 protein depletion and a reduced *SLC30A8* gene expression by 57.01% (p=0.041) ( Figure 6B and C). *KCNJ11* and *ABCC8* expression in silenced cells was as previously described, reduced by 28.23% (p=0.007) and 28.46% (p=0.011), respectively ( Figure 6C) ^34^. In *SLC30A8*-KO cells, however, *KCNJ11* and *ABCC8* mRNA levels were unchanged compared to EV control cells (104.20%, p=0.948 and 94.66%, p=0.529) ( Figure 6C).

This comparison between siRNA and KO models in EndoC-βH1 demonstrate that KO LoF models can potentiate the phenotype due to complete and permanent loss of protein, as seen in *NEUROD1*-KO cells. On the other hand, the phenotype in stable cell lines can also be diminished or not detectable at all as described in *SLC30A8*-KO cells. These additional KO cell lines show the importance of using multiple approaches to study the role of genes of interest and highlight how our KO pipeline in EndoC-βH1 cells has added an extra dimension.

## Discussion

The EndoC-βH1 cell line is an authentic human beta cell line, which is arguably the best current model to study human beta cell function. Due to its challenging growth and culture characteristics, robust protocols for CRISPR/Cas9 disease modelling to study genes implicated in human beta cell function have not been described yet in EndoC-βH1. Here, we have developed a pipeline to create stable EndoC-βH1 KO cell lines and have characterised the modifications in several proof-of-concept KO cell lines. Overall, we have successfully created five independent EndoC-βH1 KO cell lines with different gene structures, isoform expression, protein localisation and function, demonstrating that this pipeline is not restricted to certain subset of genes. The proof-of-concept cell lines, *PAM*-KO, *IDE*-KO and *INS*-KO cell lines showed an editing efficiency greater than 87.5% for each sgRNA and complete protein depletion, indicating high KO efficiency.

Editing strategies using only one sgRNA rely on a single cleavage event followed by non-homologous end-joining-mediated introduction of a frameshift mutation leading to a PSC. However, only two-thirds of frameshift mutations introduce a PSC and low sgRNA efficiencies, alternative splicing to avoid the introduced PSC and mutations escaping NMD can reduce the KO frequency ^61^. To increase the likelihood of achieving a functional KO, we implemented a strategy using two separate sgRNA ^52, 62^. Performing CRISPR/Cas9 editing with two sgRNA does not only increase the probability of creating a PSC at an individual sgRNA target site, it might also result in a large deletion by creating a pair of double-strand breaks (DSB) and thus rendering the resulting protein non-functional. In addition to the non-functional proteins, large deletions however might also result in the excision of intronic enhancers and non-coding regulatory elements. This might affect the regulation and expression of other genes and potentially introduce unintended phenotypes which could explain some of the differences between KD and KO phenotypes. In addition to the non-functional proteins, large deletions however might also result in the excision of intronic enhancers and non-coding regulatory elements. This might affect the regulation and expression of other genes and potentially introduce unintended phenotypes which could explain some of the differences between KD and KO phenotypes. Using several sgRNAs also increases the chance of off-target cleavage. Sequencing of selected high chance off-target sides in *PAM*-KO has not demonstrated any significant off-target effects. As off-target effects are sgRNA-specific, it cannot be excluded that DSBs have occurred at other sites, for any of the other sgRNAs or large deletions or rearrangements are present which exceed the range of the performed targeted PCR and sequencing reaction ^63^. Further, it might be possible that off-target activity increases during long-term culturing due to stable integration of Cas9 and the sgRNAs. We applied this dual sgRNA strategy by transducing EndoC-βH1 with individual lentivirus for each sgRNA. To obtain a more homogenous population, avoid transduction variabilities and achieve efficient dual sgRNA-based deletions, using a single expression vector containing both sgRNAs would further advance this pipeline. This can be achieved through dual-sgRNA cloning into a Cas9 containing backbone. A cost-effective and versatile protocol has recently been described and successfully been used to delete transcriptional enhancers in EndoC-βH3 ^64, 65^. EndoC-βH3 is a drug inducible conditionally immortalized human beta cell line with similar characteristics as EndoC-βH1 albeit not glucose-responsive when left untreated and only demonstrating a stable phenotype for a limited time in culture after transgene excision ^66^.

A recent study describing an *HNF1A* KO cell line in EndoC-βH1 has utilised a similar approach based on lentiviral transduction with a single sgRNA in a modified lentiCRISPRv2 vector ^40^. However, the study has not investigated if the transduction and selection process had any impact on the functionality of the cells and the resulting cell line does not demonstrate a complete protein depletion, as 10% of the cells still contain HNF1A protein. When only HNF1A negative sorted cells were studied, expression analysis could validate their findings from embryonic stem cell models and thus illustrates how KO models in EndoC-βH1 can provide relevant insights into beta cell function. Our KO models in comparison showed a complete protein depletion, which could be due to our dual sgRNA strategy, which results in a higher editing efficiency. Such a cell line with complete protein depletion makes it possible to study the KO consequences without a low level of background expression, which could mask some effects or increase the complexity of the pipeline by having to sort for complete KO cells.

Even though complete protein depletion is present in KO cells, phenotypic consequences can vary greatly compared to temporary protein reduction through siRNA-based approaches as demonstrated in assessing KO and KD strategies for *SLC30A8* and *NEUROD1*. *NEUROD1*-KO and *SLC30A8*-KO cells exhibit opposite directions of effect compared to siRNA KD. *NEUROD1*-KO show a potentiated impact on ER stress and apoptosis whereas effects on target genes in *SLC30A8*-KO are diminished. These differences are in line with previous studies demonstrating contrasting effects between KO and KD approaches ^67– 69^. The genetic compensation response, which might be masking KO mediated effects, has recently been attributed to nonsense-induced transcriptional compensation through degradation of PSC containing mRNA and should be taken into account when designing future KO sgRNA strategies ^70, 71^. Another consideration for comparing KO and KD experiments are potential seed-based, microRNA-like off-target effects from siRNA due to partial sequence complementarity to 3′ UTRs regions of other mRNA transcripts ^72^. To increase the confidence that the observed phenotype results from silencing of the intended target gene and not due to off-target effects, it is crucial to confirm the pooled siRNA approach using multiple individual siRNAs, lentiviral delivered shRNA or rescue experiments ^73, 74^. In addition, it cannot be excluded that the experimental setups including distinct reagents have contributed to the observed differences between KO and KD cells for example by inducing different levels of susceptibility to cell death.

Interestingly, EndoC-βH1 *NEUROD1*-KO cells were not viable, which is consistent with a detected increase of ER stress and apoptosis markers. In line with this, pancreases from *NeuroD1* null mice have 14-fold more apoptotic cells ^75^. Mice with conditional *NeuroD1*-KO in insulin expressing cells on the other hand do not demonstrate increased apoptosis as measured by activated caspase 3 ^58^. Overexpression of *NeuroD1* in rodent beta cell lines prevents ethanol induced expression of *Ddit3* (CHOP), reduces apoptosis and highlights *Ddit3* as a downstream target of NeuroD1 ^76^. This is in accordance with the results of both, our *NEUROD1*-KO and siRNA model which show an increase in CHOP protein, confirming the observed relation between *NEUROD1* and *DDIT3* in a human beta cell model and without extrinsic stress stimuli. Whereas temporary silencing of NEUROD1 induces a phenotype similar to chronic ER stress but without any effects on cell viability, *NEUROD1*-KO cells are not able to compensate for the permanent and complete loss of NEUROD1 protein and demonstrate elevated ER stress and apoptosis. However, further studies are needed to investigate the potential regulatory role of *NEUROD1* in ER stress and apoptosis, and its implications for diabetes.

This genome editing pipeline in EndoC-βH1 is an efficient strategy to robustly create KO cell lines in a human beta cell line. The generated KO cell lines could be used to study the function of genes in human beta cells, investigate their role in diabetes pathology and as a protein free cellular system to overexpress and study genetic variants implicated in disease. As every other LoF model, observed phenotypes might be specific to this strategy and should be validated with complementary approaches such as transient siRNA transfection. The successful generation of these KO cell lines demonstrate the feasibility of CRISPR/Cas9 genome editing in EndoC-βH1 and open up further possibilities for CRISPR/Cas9 based strategies such as CRISPR interference (CRISPRi), CRISPR activation (CRISPRa), genome-wide CRISPR screening, epigenome and base editing.

## Data availability

### Underlying data

European Nucleotide Archive: A CRISPR/Cas9 genome editing pipeline in the EndoC-βH1 cell line to study genes implicated in beta cell function. Accession number PRJEB34547; http://identifiers.org/ena.embl:PRJEB34547.

Open Science Framework: A CRISPR/Cas9 genome editing pipeline in the EndoC-βH1 cell line to study genes implicated in beta cell function. https://doi.org/10.17605/OSF.IO/2KYAN ^77^.

This project contains the following underlying data:

- Figure 1: 1F Puromycin kill curve (Raw data in an Excel file)
- Figure 2:
  ◦ 2A IDE sgRNA, 2A INS sgRNA_1D IDE KO, 2A INS sgRNA_1D INS KO, 2A PAM sgRNA, 2B Cas9, 2B Cas9_INSKO, 2B Tubulin, 2B Tubulin_INSKO and 2D PAM KO (Uncropped images in Image Lab and TIFF files)
  ◦ 2E_MED15 EV (Raw sequencing file)
  ◦ 2E_MED15 PAMKO (Raw sequencing file)
  ◦ 2F_GPM6B EV (Raw sequencing file)
  ◦ 2F_GPM6B PAMKO (Raw sequencing file)
  ◦ Figure 2C (Raw sequencing files)

- Figure 3:
  ◦ 3A Secretion data, 3B Fold change, 3C Insulin content and 3D mRNA expression (Raw data in Excel files)
  ◦ 3E IDE, 3E INS, 3E PAM, 3E Tubulin_IDE, 3E Tubulin_INS and (Uncropped images in an Image Lab and TIFF file)

- Figure 4 (All uncropped images in Image Lab and TIFF files)

- Figure 5:
  ◦ 5A GAPDH, 5A NEUROD1, 5C CHOP, 5CE TUBULIN, 5E pERK, 5G Cleaved Caspase3 and 5G TUBULIN (Uncropped images in Image Lab and TIFF files)
  ◦ 5BDFH Quantification (Raw data in an Excel file)
  ◦ 5I Cell Count (Raw data in an Excel file)
  ◦ 5J Gene expression (Raw data in an Excel file)

- Figure 6:
  ◦ 6A SLC30A8, 6A TUBULIN, 6B SLC30A8 and 6B TUBULIN (Uncropped images in Image Lab and TIFF files)
  ◦ 6C Gene expression (Raw data in an Excel file)

- Extended data:
  ◦ A GAPDH_Traf2, A Traf2, C pIre1, E Ki67 and CE Tubulin_pIre1 and Ki67 (Uncropped images in Image Lab and TIFF files)
  ◦ BDF Quantification (Raw data in an Excel file)

### Extended data

Open Science Framework: A CRISPR/Cas9 genome editing pipeline in the EndoC-βH1 cell line to study genes implicated in beta cell function. https://doi.org/10.17605/OSF.IO/2KYAN ^77^.

This project contains the following extended data:

- Extended data.tif: (A–F), Western Blot analysis in cells treated with siRNA targeting *NEUROD1* (si *NEUROD1*) compared to control siRNA (siNT). Western blots and quantification for TRAF2 (A, B), pIRE1 (C, D) and Ki67 (E, F), β-Tubulin and GAPDH are displayed as loading controls. Protein values were normalised to their respective loading controls and siNT within each experiment and fold changes are displayed as percentage of siNT, which is indicated as a dotted line at 100%. Fold changes were log-transformed for statistical analysis. P values * < 0.05, ** < 0.01 and *** < 0.001 using one-sample t-test.

Data held by Open Science Framework are available under the terms of the Creative Commons Zero "No rights reserved" data waiver (CC0 1.0 Public domain dedication).
