# Supplementary material for: A CRISPR/Cas9 genome editing pipeline in the EndoC-βH1 cell line to study genes implicated in beta cell function
Source: Wellcome Open Res. 2020 Apr 29;4:150. Originally published 2019 Oct 8. [Version 2] doi: 10.12688/wellcomeopenres.15447.2 (PMC6961417; doi:10.12688/wellcomeopenres.15447.2)
Supplement: Peer review history

# Peer review: Reviewer response for version 2

**DOI:** 10.21956/wellcomeopenres.17427.r38586

**Published:** 2020-04-30

**Authors:** Inês Cebola

**Affiliations:** 1 Section of Genetics and Genomics, Department of Metabolism, Digestion and Reproduction, Imperial College London, London, UK

Thank you for the revisions. I have no further comments to make on the manuscript.

Is the rationale for developing the new method (or application) clearly explained?

Yes

Is the description of the method technically sound?

Yes

Are the conclusions about the method and its performance adequately supported by the findings presented in the article?

Yes

If any results are presented, are all the source data underlying the results available to ensure full reproducibility?

Yes

Are sufficient details provided to allow replication of the method development and its use by others?

Yes

Reviewer Expertise:

Epigenomics, Gene regulation, CRISPR-Cas9 tools, Genetics of metabolic disorders

I confirm that I have read this submission and believe that I have an appropriate level of expertise to confirm that it is of an acceptable scientific standard.

# Peer review: Reviewer response for version 1

**DOI:** 10.21956/wellcomeopenres.16893.r37479

**Published:** 2020-01-23

**Authors:** Bridget K. Wagner

**Affiliations:** 1 Chemical Biology and Therapeutics Science Program, Broad Institute of MIT and Harvard, Cambridge, MA, USA

In this study, Grotz *et al*. have developed a method for CRISPR/Cas9-based genome editing in a human beta cell line, EndoC-bH1. The authors used three genes known to be important for beta-cell function, INS, IDE, and PAM, to validate the method. Further, they employ a dual-sgRNA strategy and high MOI, to increase the likelihood of transducing cells with both guides and to increase the likelihood of knocking out the gene of interest.

 They first demonstrate that introduction of Cas9 to the cells does not interfere with glucose-stimulated insulin secretion. The genes of interest were also verified to be knocked out. The KO cells were then compared to siRNA-mediated silencing, and the authors found differences in gene expression and sensitivity to apoptosis, suggesting that KO brings additional information to the study of genes of interest, and that multiple approaches should probably be taken to understand the biology.

 Overall, this study is solidly performed, and the authors have been careful in their use of the EndoC cells and in the design of sgRNAs. I had a few questions regarding the comparison of KO with KD. First, the EV cells showed quite a high level of CHOP (Fig. 4E), even though the KO increases that somewhat. Does Cas9 expression induce UPR as well? On the other hand, this was true of the siNT cells as well (Fig. 5C), so is it possible the EndoC cells themselves were under stress, even in basal conditions?

 Second, it would be nice to see a gene-expression analysis comparing NEUROD1-KO with KD, similar to that done for SLC30A8 (Fig. 6C).

 Finally, could the differences between KO and KD cells be explained by seed effects in the siRNA treatment, especially since a pool of siRNAs was used?

 Most of these questions can be addressed by editing some of the text.

Is the rationale for developing the new method (or application) clearly explained?

Yes

Is the description of the method technically sound?

Yes

Are the conclusions about the method and its performance adequately supported by the findings presented in the article?

Yes

If any results are presented, are all the source data underlying the results available to ensure full reproducibility?

Yes

Are sufficient details provided to allow replication of the method development and its use by others?

Yes

Reviewer Expertise:

Beta cell biology, chemical biology.

I confirm that I have read this submission and believe that I have an appropriate level of expertise to confirm that it is of an acceptable scientific standard.

# Response

**Published:** 2020-04-21

**Authors:** Antje Grotz

**Affiliations:** University of Oxford, UK

*In this study, Grotz et al. have developed a method for CRISPR/Cas9-based genome editing in a human beta cell line, EndoC-bH1. The authors used three genes known to be important for beta-cell function, INS, IDE, and PAM, to validate the method. Further, they employ a dual-sgRNA strategy and high MOI, to increase the likelihood of transducing cells with both guides and to increase the likelihood of knocking out the gene of interest.*

*They first demonstrate that introduction of Cas9 to the cells does not interfere with glucose-stimulated insulin secretion. The genes of interest were also verified to be knocked out. The KO cells were then compared to siRNA-mediated silencing, and the authors found differences in gene expression and sensitivity to apoptosis, suggesting that KO brings additional information to the study of genes of interest, and that multiple approaches should probably be taken to understand the biology.*

*1. Overall, this study is solidly performed, and the authors have been careful in their use of the EndoC cells and in the design of sgRNAs. I had a few questions regarding the comparison of KO with KD. First, the EV cells showed quite a high level of CHOP (Fig. 4E), even though the KO increases that somewhat. Does Cas9 expression induce UPR as well? On the other hand, this was true of the siNT cells as well (Fig. 5C), so is it possible the EndoC cells themselves were under stress, even in basal conditions?*

First of all, we would like to thank you for taking the time to review this manuscript and for the constructive and helpful feedback. Cas9 expression does not induce UPR which we assessed by comparing basal levels of CHOP in WT (no Cas9) and EV (Cas9 only) cells. We clarified this in the main text by adding: ‘There was no difference in ER stress or apoptotic markers between WT and EV cells (not shown)’.

EndoC-βH1 cells have high basal levels of ER stress which could be due to high baseline ER stress which is observed in beta-cells as a result of their function as secretory cells and the associated insulin production. However, we have not compared their ER stress levels to other models of beta-cells and therefore have no indication if the levels are even further increased. In line with our results about basal levels of high ER stress in EndoC-βH1, Oleson et al demonstrated the same observation for markers such as cleaved caspase 3 or HSP70 ^1^. As the high levels of CHOP have been consistent across experiments and researchers and can be induced even further, we can assume that the cells were not stressed beyond their baseline.

*2. Second, it would be nice to see a gene-expression analysis comparing NEUROD1-KO with KD, similar to that done for SLC30A8 (Fig. 6C).*

Thank you for this suggestion, we agree that it would have been interesting to explore this aspect. However, due to the ongoing cell death and resulting very low cell numbers of *NEUROD1*-KO cells, we were unable to collect sufficient cells for both, protein and RNA analysis. We therefore decided to prioritise analysing the differences in protein levels between *NEUROD1*-KO and EV cells for validated markers of UPR activation and ER stress.

*3. Finally, could the differences between KO and KD cells be explained by seed effects in the siRNA treatment, especially since a pool of siRNAs was used?*

We agree that this is an important aspect to be considered which we have not discussed sufficiently. We have now addressed this in the discussion section of the revised manuscript: ‘Another consideration for comparing KO and KD experiments are potential seed-based, microRNA-like off-target effects from siRNA due to partial sequence complementarity to 3′ UTRs regions of other mRNA transcripts ^2^. To increase the confidence that the observed phenotype results from silencing of the intended target gene and not due to off-target effects, it is crucial to confirm the pooled siRNA approach using multiple individual siRNAs, lentiviral delivered shRNA or rescue experiments ^3,4^.’

*Most of these questions can be addressed by editing some of the text.*

1. Oleson, B. J. *et al.* Distinct differences in the responses of the human pancreatic β-cell line endoc-βh1 and human islets to proinflammatory cytokines. *Am. J. Physiol. - Regul. Integr. Comp. Physiol.* **309**, R525–R534 (2015).

2. Doench, J. G., Petersen, C. P. & Sharp, P. A. siRNAs can function as miRNAs. *Genes Dev.* **17**, 438–442 (2003).

3. Klinghoffer, R. A. *et al.* Reduced seed region-based off-target activity with lentivirus-mediated RNAi. *Rna* **16**, 879–884 (2010).

4. Jackson, A. L. & Linsley, P. S. Recognizing and avoiding siRNA off-target effects for target identification and therapeutic application. *Nat. Rev. Drug Discov.* **9**, 57–67 (2010).

# Peer review: Reviewer response for version 1

**DOI:** 10.21956/wellcomeopenres.16893.r37481

**Published:** 2020-01-13

**Authors:** Inês Cebola

**Affiliations:** 1 Section of Genetics and Genomics, Department of Metabolism, Digestion and Reproduction, Imperial College London, London, UK

In this work, Grotz *et al.* develop a robust method to generate human beta cell lines harbouring gene KOs with high efficiency. A fact that perhaps will be elusive to those from outside the pancreatic β cell field is that cells from the EndoC βH family grow slowly and very sensitive to culture conditions. Consequently, it would be extremely challenging to generate clonal populations with these cells as it is normally done in the genome editing field, particularly by those working with stem cells or cancer cell lines. Even so, EndoC βH cells are currently a gold standard human beta cell model, when studies on human tissue are impractical, and therefore efforts to develop new methodologies around them are welcomed by the community.

 The work scientifically sound and I praise the authors for the detailed description of the methods and results. Below I offer minor suggestions for improvement, which the authors may want to address to improve the manuscript further:

  

- The authors set the tone very well in the introduction. In addition to the functional features that the authors already describe, I would only suggest adding a short reference to previous work demonstrating the utility of EndoC βH1 cells to model transcriptional and epigenomic features, given their resemblance with primary human beta cells (Lawlor *et al.* Cell Reports 2019) ^1^.
- The western blot data showing complete gene KO is very convincing. It would be interesting to know how stable these KO lines are, especially after the observations made by the authors in Figure 4B, comparing NEUROD1 KO at weeks 1 and 2. How long did the authors maintain the other KO lines in culture? This information could be added in the main text and/or in the figure legends.
- Regarding the design, a few additional clarifications could have been provided to add even more value to the pipeline. In particular, the authors mention that two sgRNAs were selected per gene, based on highest specificity and lowest off-target score. Did the authors also require that the expected excised fragment yields a gene KO (i.e. that the length of the excised coding sequence is not a multiple of three)? If this was the case, as it seems from the *INS* example, it should be stated in the manuscript.
- While for the *INS* gene, the authors produced KO cells carrying a 55 bp deletion, for the other examples, the authors designed KOs of several kbs. The reasoning to choose specific sgRNAs for the targeting is sound (highest predicted specificity and lowest off-targeting). Nevertheless, deleting large fragments may produce undesired results such as the KO of intronic enhancers and other noncoding regulatory elements, which may be involved in the regulation of other genes. Could this somehow affect the results observed with these KO lines, especially in the comparisons between KO and KD? The authors should at least discuss this possibility.
- The comparison between KO and KD cells is very interesting conceptually. It is debatable however whether the results are directly comparable given the fact that factors such as time after induction of loss-of-function, delivery method (lentiviral vs. transfection), molecular machinery deployed and associated cytotoxicity are distinct between the two approaches. Could these experimental setup factors contribute to differential susceptibility to cell death ( *NEUROD1*) or transcriptional dysregulation ( *SLC30A8*)? The authors could explore these possibilities further in the discussion. Still, what these results point out is that multiple approaches are necessary to drill into the molecular mechanism of a particular locus and that there is no “one-fits-all” solution. This is an important point that the authors transmit well.

 

*Other considerations:*

 These two types of experiment (siRNA and CRISPR KO) are often applied and interpreted as if they were the same, when in fact the authors observed different results between KO and KD, where residual protein could still be present. It seems logical that a KO will produce a more severe phenotype than a transient KD of the same gene, as the authors observe with *NEUROD1* (Figure 5). In Figure 6, however, the authors observe the opposite trend, with the gene KD affecting the expression of the two genes encoding the subunits of a pancreatic β cell potassium channel, but not the KO. Previous work by the Gloyn group established that not only *SLC30A8 *KD, but also presence of specific variants in *SLC30A8* associated with less transcription of the gene, were linked with decreased *KCNJ11* and *ABCC8* expression. Moreover, human islet data revealed correlation between mRNA levels of *SLC30A8 *and the other two genes. The observations with the lentiCRISPR-mediated KO are thus very puzzling. They are however in line with previous observations of genetic compensation, as explained by the authors. Given the clinical importance of *ABCC8*, the results of this study invite further work to identify the genes that may be compensatory for *ABCC8* loss. In my opinion however, such studies would be outside the scope of this work.

 In summary, this work provides a straightforward approach to generate LoF human pancreatic β cell models within a reasonable timeframe for mechanistic investigations. As with any method, its applicability is limited and additional methods should be applied to fully characterise genetic loci of interest.

Is the rationale for developing the new method (or application) clearly explained?

Yes

Is the description of the method technically sound?

Yes

Are the conclusions about the method and its performance adequately supported by the findings presented in the article?

Yes

If any results are presented, are all the source data underlying the results available to ensure full reproducibility?

Yes

Are sufficient details provided to allow replication of the method development and its use by others?

Yes

Reviewer Expertise:

Epigenomics, Gene regulation, CRISPR-Cas9 tools, Genetics of metabolic disorders

I confirm that I have read this submission and believe that I have an appropriate level of expertise to confirm that it is of an acceptable scientific standard.

## References

[1] : Multiomic Profiling Identifies cis-Regulatory Networks Underlying Human Pancreatic β Cell Identity and Function. *Cell Reports*.2019;26(3) : 10.1016/j.celrep.2018.12.083 788-801.e6 10.1016/j.celrep.2018.12.083 PMC638926930650367

# Response

**Published:** 2020-04-21

**Authors:** Antje Grotz

**Affiliations:** University of Oxford, UK

*In this work, Grotz et al. develop a robust method to generate human beta cell lines harbouring gene KOs with high efficiency. A fact that perhaps will be elusive to those from outside the pancreatic β cell field is that cells from the EndoC βH family grow slowly and very sensitive to culture conditions. Consequently, it would be extremely challenging to generate clonal populations with these cells as it is normally done in the genome editing field, particularly by those working with stem cells or cancer cell lines. Even so, EndoC βH cells are currently a gold standard human beta cell model, when studies on human tissue are impractical, and therefore efforts to develop new methodologies around them are welcomed by the community.*

*The work scientifically sound and I praise the authors for the detailed description of the methods and results. Below I offer minor suggestions for improvement, which the authors may want to address to improve the manuscript further:*

**

*1. The authors set the tone very well in the introduction. In addition to the functional features that the authors already describe, I would only suggest adding a short reference to previous work demonstrating the utility of EndoC βH1 cells to model transcriptional and epigenomic features, given their resemblance with primary human beta cells (Lawlor et al. Cell Reports 2019)* *^1^* *.*

We would like to thank you first for taking the time to review this manuscript and for the thoughtful and constructive feedback. We agree that this is an important and relevant publication which readers will find useful and we have therefore added the following paragraph in the introduction: ‘Multiomic profiling in EndoC-βH1 cells including epigenomic and transcriptomic maps largely recapitulate primary human islets signatures and along with their similar electrophysiological properties, EndoC-βH1 are therefore a representative model of human beta cells and physiological insulin secretion ^28, 29, 30^.’

*2. The western blot data showing complete gene KO is very convincing. It would be interesting to know how stable these KO lines are, especially after the observations made by the authors in Figure 4B, comparing NEUROD1 KO at weeks 1 and 2. How long did the authors maintain the other KO lines in culture? This information could be added in the main text and/or in the figure legends.*

Thank you for pointing this out, we agree that this important information had been missing and we added the following sentence ‘The KO cell lines were stably cultured for more than six months without losing their KO phenotype.’

*3. Regarding the design, a few additional clarifications could have been provided to add even more value to the pipeline. In particular, the authors mention that two sgRNAs were selected per gene, based on highest specificity and lowest off-target score. Did the authors also require that the expected excised fragment yields a gene KO (i.e. that the length of the excised coding sequence is not a multiple of three)? If this was the case, as it seems from the INS example, it should be stated in the manuscript.*

We agree that this would be an additional criterion to ensure gene KO, however we did not specifically consider this in our current sgRNA design but we would recommend to incorporate it in future KO pipelines.

*4. While for the INS gene, the authors produced KO cells carrying a 55 bp deletion, for the other examples, the authors designed KOs of several kbs. The reasoning to choose specific sgRNAs for the targeting is sound (highest predicted specificity and lowest off-targeting). Nevertheless, deleting large fragments may produce undesired results such as the KO of intronic enhancers and other noncoding regulatory elements, which may be involved in the regulation of other genes. Could this somehow affect the results observed with these KO lines, especially in the comparisons between KO and KD? The authors should at least discuss this possibility.*

Thank you for this excellent suggestion, we have added the following paragraph to address this: ‘In addition to the non-functional proteins, large deletions however might also result in the excision of intronic enhancers and non-coding regulatory elements. This might affect the regulation and expression of other genes and potentially introduce unintended phenotypes which could explain some of the differences between KD and KO phenotypes.’

*5. The comparison between KO and KD cells is very interesting conceptually. It is debatable however whether the results are directly comparable given the fact that factors such as time after induction of loss-of-function, delivery method (lentiviral vs. transfection), molecular machinery deployed and associated cytotoxicity are distinct between the two approaches. Could these experimental setup factors contribute to differential susceptibility to cell death (NEUROD1) or transcriptional dysregulation (SLC30A8)? The authors could explore these possibilities further in the discussion. Still, what these results point out is that multiple approaches are necessary to drill into the molecular mechanism of a particular locus and that there is no “one-fits-all” solution. This is an important point that the authors transmit well.*

You raise an important point and we agree that many factors could play a role in the observed differences, we have therefore added the following sentence in the discussion to emphasise this point: ‘In addition, it cannot be excluded that the experimental setups including distinct reagents have contributed to the observed differences between KO and KD cells for example by inducing different levels of susceptibility to cell death.’

# Peer review: Reviewer response for version 1

**DOI:** 10.21956/wellcomeopenres.16893.r36820

**Published:** 2019-11-11

**Authors:** Shuibing Chen

**Affiliations:** 1 Department of Biochemistry, Weill Cornell Medical College, New York, NY, USA

In this manuscript, Grotz *et al.* reported using stable CRISPR/Cas9 KO in EndoC-βH1 cells to study the role of beta cell key regulators in beta cell survival *. *First, sequencing and western blotting were used to validate the KO efficiency. Then, the authors compared the KO cell lines siRNA-mediated knockdown (KD) approaches and found different phentypes. *NEUROD1-*KO cells were not viable and displayed elevated markers for ER stress and apoptosis.

*NEUROD1*-KD, however, only had a modest elevation of CHOP without evidence of elevated cell death. In addition, *SLC30A8*-KO cells demonstrated no reduction in K channel gene expression in contrast to siRNA silencing. The manuscript reported a useful platform to study the role of beta cell regulators in beta cell function and survival. However, additional work need to be done to be considered for indexing.

 Major issues:

 a. Several genes KO in this study are key beta cell regulators, such as *INS*, *IDE*, *PAM, NEUROD1, *and *SLC30A8. *However, the characterization of the KO cells are very limited. Please systematically examine:

1. The key markers of beta cells.
2. Total insulin content.
3. Beta cell function. 

b. Additional experiments are needed to explain the difference between KO and KD lines. It is due to the remaining dose of the protein in KD lines. If yes, an inducible shRNA system might be required to study the dose-dependent activity of NEUROD1 and SLC30A8.

Is the rationale for developing the new method (or application) clearly explained?

Partly

Is the description of the method technically sound?

Yes

Are the conclusions about the method and its performance adequately supported by the findings presented in the article?

Partly

If any results are presented, are all the source data underlying the results available to ensure full reproducibility?

Partly

Are sufficient details provided to allow replication of the method development and its use by others?

Yes

Reviewer Expertise:

Beta cell biology and gene knockout.

I confirm that I have read this submission and believe that I have an appropriate level of expertise to confirm that it is of an acceptable scientific standard, however I have significant reservations, as outlined above.

# Response

**Published:** 2020-04-21

**Authors:** Antje Grotz

**Affiliations:** University of Oxford, UK

*1. In this manuscript, Grotz et al. reported using stable CRISPR/Cas9 KO in EndoC-βH1 cells to study the role of beta cell key regulators in beta cell survival. First, sequencing and western blotting were used to validate the KO efficiency. Then, the authors compared the KO cell lines siRNA-mediated knockdown (KD) approaches and found different phentypes. NEUROD1-KO cells were not viable and displayed elevated markers for ER stress and apoptosis.*

*NEUROD1-KD, however, only had a modest elevation of CHOP without evidence of elevated cell death. In addition, SLC30A8-KO cells demonstrated no reduction in K channel gene expression in contrast to siRNA silencing. The manuscript reported a useful platform to study the role of beta cell regulators in beta cell function and survival. However, additional work need to be done to be considered for indexing.*

First, we would like to thank you for the thoughtful feedback and for taking the time to review this manuscript. We have addressed your concerns with detailed comments below and hope this will clarify the intended aims of our paper. We would like to stress that our objective is to provide a methodological paper detailing a protocol which we feel is a valuable resource for the community in as timely a manner as possible without performing additional in-depth functional follow-up work. We now make this point clearer in our revised manuscript so that readers can understand that the characterisation of the cell lines is not the main objective of the study and consequently our characterisation is not comprehensive. We have added the following sentence in the introduction ‘This CRISPR/Cas9 pipeline and the resulting KO cell lines could be a valuable tool in understanding human beta cell function and genes underlying the pathology for both T2D and T1D.’

*2. Major issues:*

*a. Several genes KO in this study are key beta cell regulators, such as INS, IDE, PAM, NEUROD1, and SLC30A8. However, the characterization of the KO cells are very limited. Please systematically examine: *

1. *The key markers of beta cells.*
2. *Total insulin content.*
3. *Beta cell function.*

It would certainly be interesting and valuable to systematically characterise the *INS*, *IDE*, *PAM, NEUROD1, *and *SLC30A8* KO cell lines. However, the aim of our manuscript was to provide details of our pipeline to generate CRISPR KOs in this human beta cell line and not to characterise the role of these genes in human beta-cell function. Our goal was to provide details of our pipeline for those wishing to generate their own KO-cell lines and not to characterise these genes. We feel that these additional studies are beyond the scope of this manuscript.

*3. Additional experiments are needed to explain the difference between KO and KD lines. It is due to the remaining dose of the protein in KD lines. If yes, an inducible shRNA system might be required to study the dose-dependent activity of NEUROD1 and SLC30A8.*

Again, we agree that the difference in cellular phenotype between these two experimental approaches is fascinating and future studies should indeed explore the relationship between transcript expression levels and phenotype. There are many potential explanations for this phenomenon (such as transcriptional adaptation as discussed in Ref 69 and 70) and addressing this comprehensively would require a substantial body of additional work which is in our opinion, beyond the scope of this methodological report. To reiterate our aim with this methodological report was to provide details of how to create KO cell models in this line and not to determine the differences between KD and KO loss-of-function models or dose-dependent activities of NEUROD1 and SLC30A8.

*4.* ***If any results are presented, are all the source data underlying the results available to ensure full reproducibility?*** *Partly*

We would also like to note that all underlying source data has been made available at Open Science Framework in cooperation with the editors as explained in the ‘Data availability’ section.

# Peer review: Reviewer response for version 1

**DOI:** 10.21956/wellcomeopenres.16893.r36779

**Published:** 2019-11-01

**Authors:** Hindrik Mulder

**Affiliations:** 1 Department of Clinical Sciences in Malmö, Unit of Molecular Metabolism, Lund University Diabetes Centre, Malmö University Hospital, Skåne University Hospital, Lund University, Lund, Sweden

Professor Anna Gloyn and co-workers present a newly developed pipeline for manipulation of gene expression in insulin-secreting cells. This work has been prompted by the need of human cells to understand beta-cell biology and the expanding availability of tools for genetic manipulation. Thus, the CRISPR-Cas9 technology, where guide RNAs specifically target DNA-sequences followed by Cas9-induced nuclease activity with ensuing DNA breaks, allows highly specific manipulation of cellular DNA with extraordinarily little effort, if comparing with existing technologies. With these components, the authors have constructed an impressive methodology to manipulate gene expression in the EndoC-betaH1 cell line. The EndoC-betaH1 cells, which were derived from human embryonic cells and are precursors of beta-cells, are not faithful counterparts of human beta-cells but have the added benefit of harbouring the human genetic machinery. This allows analysis of genetically-based disease processes in humans, which would be more difficult in rodent-derived cells.

 The authors have applied their approach to five target genes. For two of them, the results are compared with cells where the same gene has been silenced by RNA interference. The essential aspect of their approach is that two instead of the customary one guide RNAs are used. It is argued, and largely shown, that this will result in a more “controlled” and effective knock out of the targeted genes: it is intended that sequence between the sites where the guide RNAs bind will be excised. All the components of the system are cloned into a lentiviral vector, since it is known to be reasonably effective in EndoC-betaH1 cells. A potential caveat of this approach is that that the lentivirus will integrate into the host genome, which in itself may have an impact on the cells. On the other hand, a stable transduction will be achieved. This can be checked by sequencing. Indeed, the authors have looked for off-target effects, also from Cas9-mediated nuclease activity, but find none. An alternative approach would have been to include a Piggybac sequence in the construct, which could be excised, and hence prevent scarring of the host DNA, or use adenoviruses, which do not integrate.

 As the authors describe, no clonal selection of the transduced EndoC-betaH1 cells was made. Hence, the transduced and edited cells will be made out of a heterogenous population of cells with long and short excisions. Nevertheless, a PCR-based analysis of the INS-KO cells show that a major proportion of transduced cells have a major part excised of the sequence between the sites where the guide RNAs have cut. This notwithstanding, the somewhat inconsistent functional and expression results that were found may be due to this heterogeneity of editing.    

 The analyses of the cells are mostly derived from expression analyses (QPCR and western blots). The only analysis of secretion is a comparison of cells transduced with empty vector and wild type cells. Although there is quite high variability in secretion, which is not strongly stimulated other than by tolbutamide, no differences between the manipulations are seen, suggesting that the molecular manipulations leave the secretion machinery largely intact.

 A very interesting and thought-provoking aspect of the work is the comparisons of knock out and silencing approaches to target the same gene; the latter has become a well-established approach in cell biology. It is thus surprising that the results, using these two approaches are so distinct and variable. Knock out of NEUROD1 resulted in cell death, inferred from the fact that knock out cells are largely absent after a few days, and instead proliferation of cells in which gene targeting has not occurred. In contrast, efficient silencing of NEUROD1 does not induce apoptosis. For manipulation of SLC30A8, expression of KCNJ11 and ABCC8 mRNA was used as read outs. Equally puzzling, levels of these important genes were lowered in silenced cells but unaffected in knock out cells. While the present work points out the problem it makes no attempts to explain it. Although it seems that deletion and silencing of the target genes and their encoded proteins have been comparable, the impact on phenotype has been variable. Clearly, there are many aspects of silencing and knock out (CRISPR) which are different: onset, extent, vector, molecular mode of action, toxicity, off-target effects, durability and more. An important lesson from this paper is that all approaches, as wonderful and effective they may seem, have limitations, and this should always be borne in mind when drawing conclusions from research.

 Another reflection from my part is that the methodological development of the genome editing techniques is astounding. Just the other week, prime-editing was published. Here addition of reverse transcript and an RNA template to the nickase affords unprecedented accuracy in single base substitutions as well as deletion of sequences of choice. Thus, the approach described here has already been surpassed by a, seemingly, superior approach. Again, this underscores that addressing research questions may be more durable than publishing methodology. Moreover, the single base approach is really what is needed in functional cellular genomics, where the impact of SNPs in risk alleles for complex diseases is analysed. The pipeline described here does not address this issue.   

 In sum, the work from Anna Gloyn’s group has introduced a novel and robust way of manipulating gene expression in highly differentiated insulin-secreting cells. It is also a cautionary tale – understand the limitations of your methods when interpreting your results.

Is the rationale for developing the new method (or application) clearly explained?

Yes

Is the description of the method technically sound?

Yes

Are the conclusions about the method and its performance adequately supported by the findings presented in the article?

Yes

If any results are presented, are all the source data underlying the results available to ensure full reproducibility?

Yes

Are sufficient details provided to allow replication of the method development and its use by others?

Yes

Reviewer Expertise:

Islet and diabetes research; metabolism

I confirm that I have read this submission and believe that I have an appropriate level of expertise to confirm that it is of an acceptable scientific standard.
